# Intra-cholecystic Tubulopapillary Neoplasm: Is Simple Cholecystectomy Enough?

**DOI:** 10.7759/cureus.93535

**Published:** 2025-09-30

**Authors:** Rahul Khullar, Hardik Parmar, Anu Shibu Varghese, Anbalagan Pillai, Suzanne Prasad

**Affiliations:** 1 Gastrointestinal Surgery, Medeor 24x7 Hospital, Abu Dhabi, ARE; 2 Gastroenterology, Medeor 24x7 Hospital, Abu Dhabi, ARE; 3 Pathology, Medeor 24x7 Hospital, Abu Dhabi, ARE; 4 Management, Medeor 24x7 Hospital, Abu Dhabi, ARE; 5 Anaesthesia, Medeor 24x7 Hospital, Abu Dhabi, ARE

**Keywords:** gall bladder malignancy, gall bladder mass, gall bladder polyp, intracholecystic papillary neoplasm, laparoscopic cholecystectomy

## Abstract

Intracholecystic tubulopapillary neoplasm (ICPN) is a noninvasive, intraepithelial tumor presenting as a polypoid papillary mass arising from the gallbladder. Approximately 6.4% of all gallbladder neoplasms are associated with ICPNs.

A young male presented with pain in the right hypochondrium and epigastrium. An abdominal ultrasound showed a 2.3 x 1.6 cm mass lesion in the gallbladder. Computed tomography (CT) of the abdomen showed an irregular polypoidal intraluminal soft tissue density of 21 x 17 mm in the fundus of the gallbladder. Endoscopic ultrasound (EUS) evaluation was suggestive of a gallbladder mass. On suspicion of carcinoma of the gallbladder, the patient underwent radical cholecystectomy with a 2 cm liver wedge with standard hepatoduodenal ligament, periportal, and retropancreatic lymphadenectomy with uneventful recovery. Postoperative histopathology revealed an ICPN with predominant intestinal morphology and low-grade dysplasia. All 16 lymph nodes were negative for malignancy. Dysplasia was present in the neck region but not in the cystic duct margin.

ICPN is a rare entity to be recognized preoperatively. It is considered the counterpart of intraductal papillary mucinous neoplasm in the pancreatic cancer spectrum. Invasive carcinoma is present in almost half of the cases. It has a propensity for rapid conversion to carcinoma of the gallbladder. Preoperatively, if suspected, it should be treated with radical cholecystectomy for the potential of a cure for the invasive component as well as for staging purposes.

## Introduction

Intracholecystic tubulopapillary neoplasm (ICPN) is a pre-invasive neoplastic lesion characterized by papillary growth in the gallbladder. ICPN can also be considered a counterpart of intraductal papillary mucinous neoplasm (IPMN) in pancreatic duct epithelium [1]. ICPN has four subtypes: gastric, biliary, intestinal, and oncocytic. Biliary and intestinal subtypes are more associated with invasion as compared to other subtypes [2]. Most of the early cases were diagnosed after routine laparoscopic cholecystectomies, and the disease entity was considered to be benign. In the 2019 WHO classification, it was proposed as a preinvasive neoplasm of the gallbladder. A case report by Suda et al. has shown the aggressive nature of these preinvasive neoplasms, especially those associated with an invasive component, to the extent of the presence of liver metastasis in a patient [3]. In light of recent events, a simple laparoscopic cholecystectomy could not justify the appropriate treatment option in this entity if it is suspected preoperatively [3,4]. Here, we present a case of a young man diagnosed with ICPN.

## Case presentation

**Figure 1:**
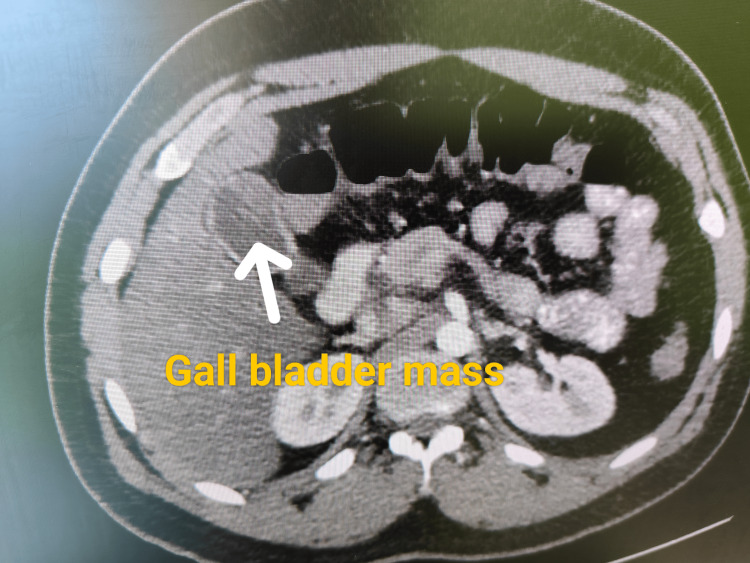
Computed tomography (CT) scan of the abdomen White arrow showing the gallbladder mass

A young male, 23 years old, presented with pain in the right hypochondrium and epigastrium, with no history of loss of appetite or loss of weight. The abdominal examination was unremarkable. Vitals and laboratory investigations were normal. The tumor marker carcinoembryonic antigen (CEA) was 1.6 ng/ml, and cancer antigen (CA19-9) was 17.9 U/ml. An abdominal ultrasound was done for abdominal pain, which showed a 2.3 x 1.6 cm mass lesion in the gallbladder. The common bile duct (CBD) was normal. Computed tomography (CT) of the abdomen showed an irregular polypoidal intraluminal soft tissue density of 21 x 17 mm in the fundus of the gallbladder (Figure 1). No other lesion or significant lymphadenopathy was noted. Endoscopic ultrasound (EUS) evaluation was suggestive of a gallbladder mass. Fine-needle aspiration (FNA)was not done to prevent tumor seeding.

**Figure 2:**
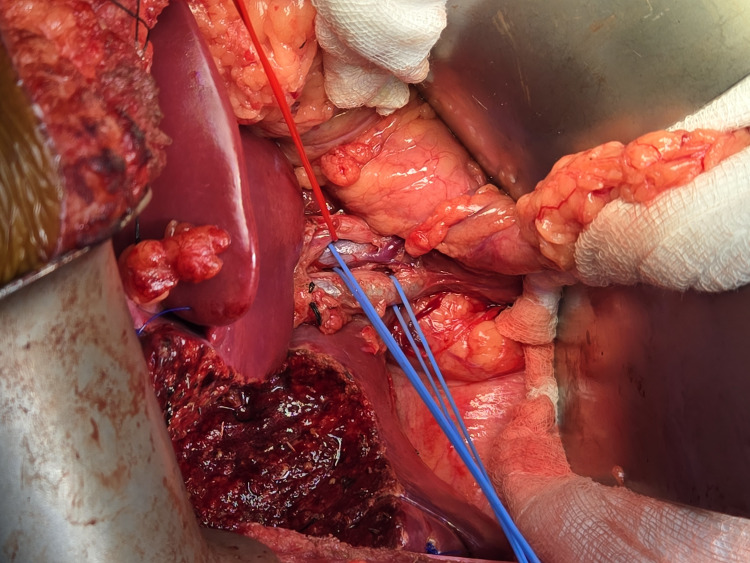
The resected gallbladder mass with a 2 cm liver wedge with complete hepatoduodenal and retropancreatic lymphadenectomy A red vascular loop in the figure encircling the hepatic artery proper, and two blue vascular loops in the figure encircling the common bile duct (CBD) and portal vein

**Figure 3:**
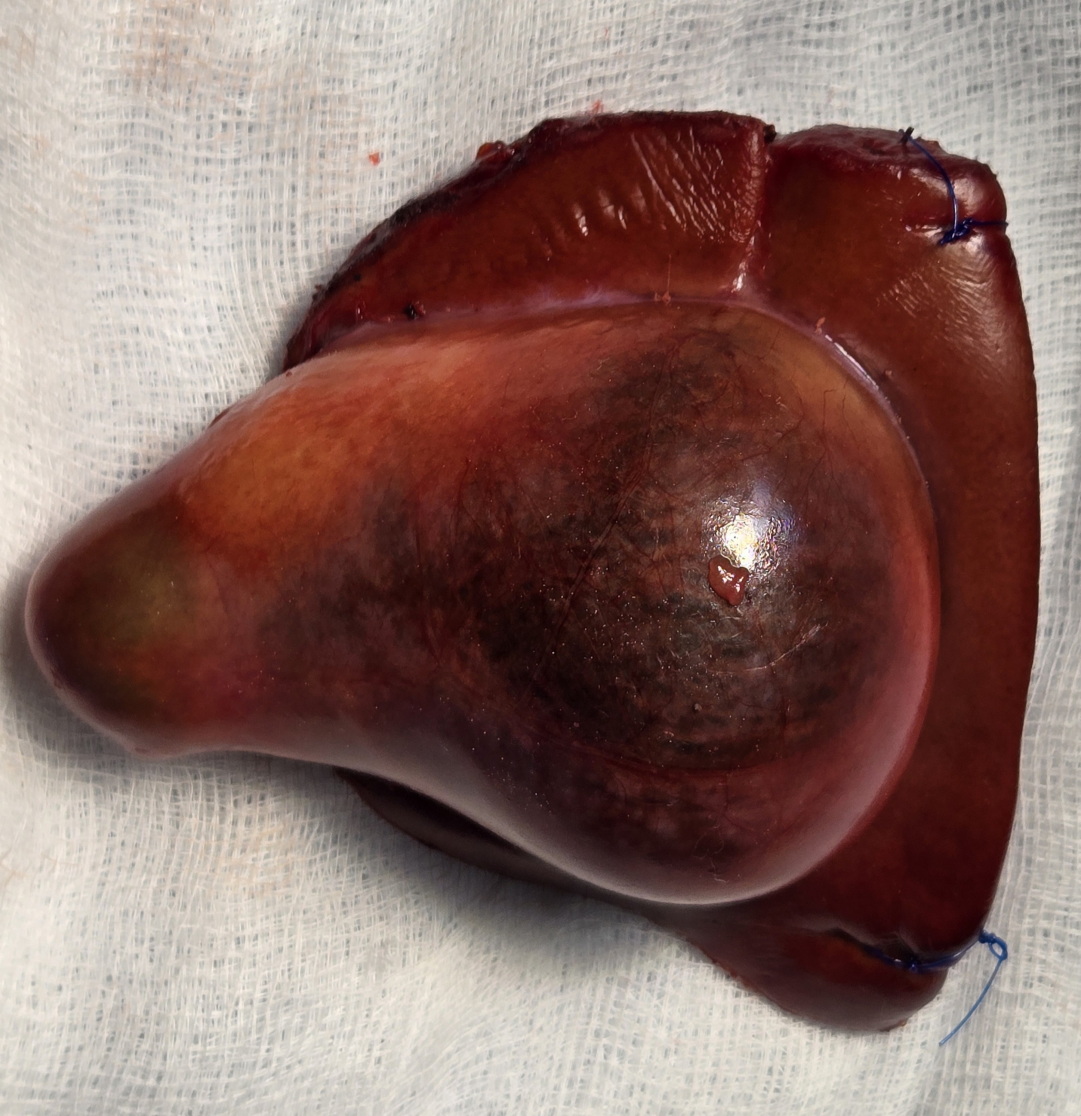
Resected specimen showing the gallbladder mass with the liver wedge

On suspicion of a mass in the gallbladder (carcinoma of the gallbladder), the patient underwent a radical cholecystectomy with a 2 cm liver wedge with a standard hepatoduodenal ligament, and periportal and retropancreatic lymphadenectomy with uneventful recovery (Figure 2 and Figure 3). The intraoperative cystic duct margin and inter-aortocaval lymph node were sent for frozen section, which was negative. The gallbladder frozen section was not sent, as malignancy in the gallbladder was suspected preoperatively. The patient tolerated the procedure well. Oral intake was allowed on postoperative day 1. On postoperative day 2, the subhepatic drain was removed, and the patient was discharged on postoperative day 4.

**Figure 4:**
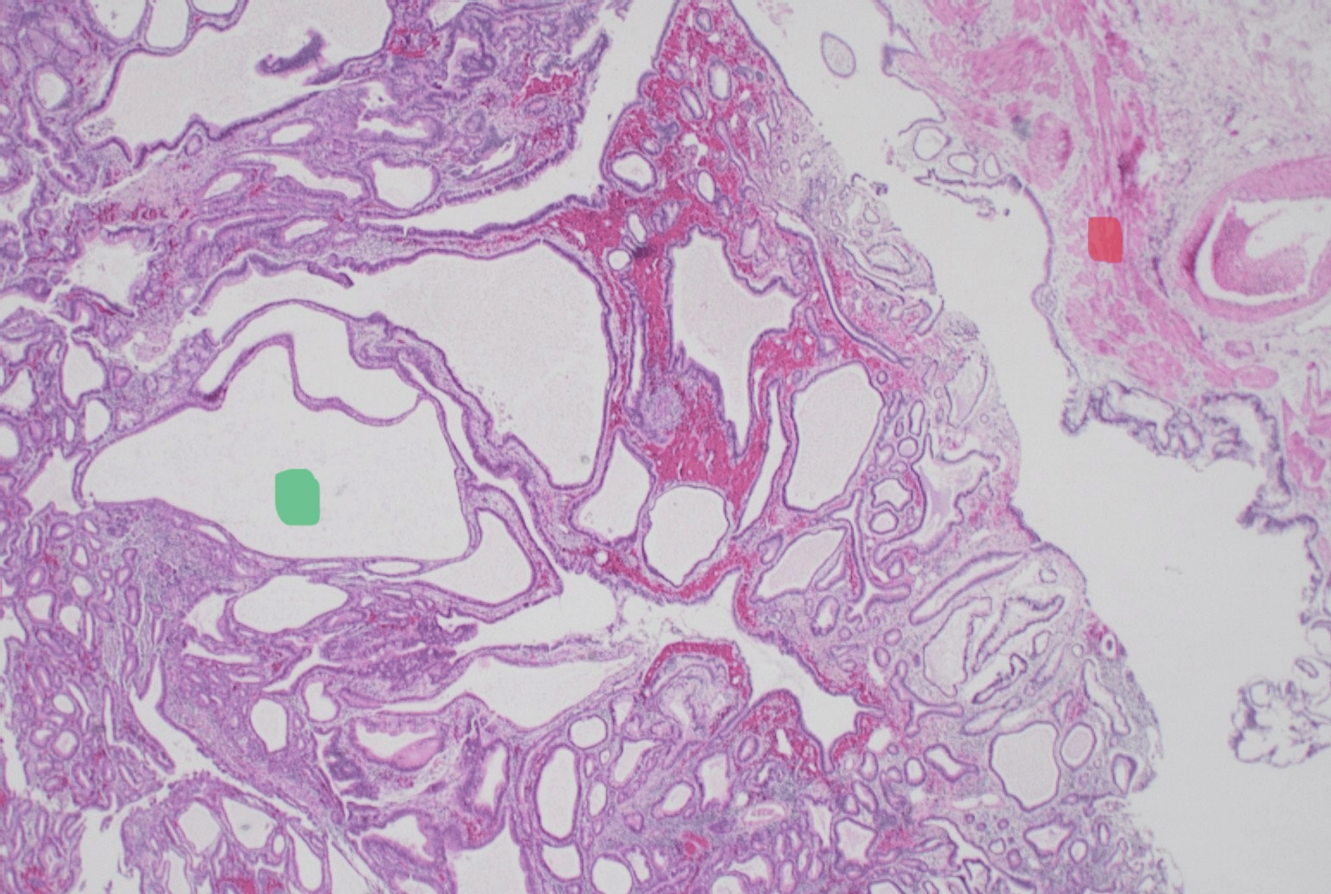
Hematoxylin and Eosin stain, 1.25x magnification The wall of the gallbladder along the right upper corner (red dot) with its muscle layer and the intraluminal polypoid tubulopapillary neoplasm (green dot)

**Figure 5:**
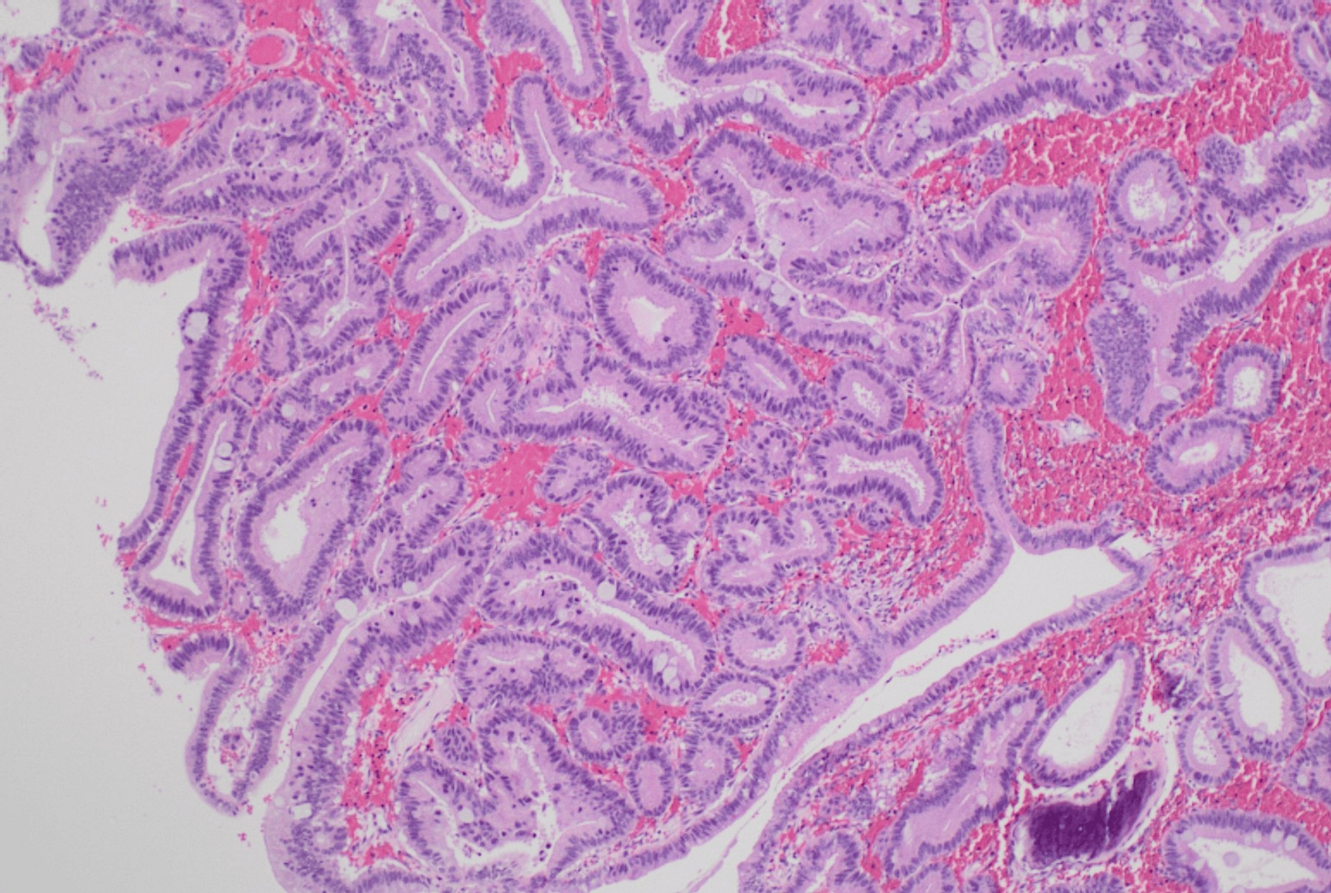
Hematoxylin and Eosin stain, 4x magnification Intracholecystic tubulopapillary neoplasm (ICPN) lined by columnar cells displaying elongated, mildly enlarged, pseudostratified, basally placed nuclei with inconspicuous nucleoli and evidence of intestinal metaplasia

Postoperative histopathology (Figure 4: Hematoxylin & Eosin stain, 1.25x magnification, and Figure 5: Hematoxylin & Eosin stain, 4x magnification) showed an intracholecystic tubulopapillary neoplasm with intestinal metaplasia and low-grade dysplasia in the neck region of the gallbladder. All 16 lymph nodes were negative for malignancy. There was no invasive component. The patient was doing well at the first follow-up six months after surgery, with a plan for tumor markers (CEA and CA 19-9) and magnetic resonance imaging (MRI) of the abdomen to be done one year after surgery. If normal, yearly follow-up is advised by the oncologist for five years.

## Discussion

ICPN, being a pre-invasive lesion, supposedly has a good prognosis with a 3-year survival of 90% for patients without an invasive component on histopathology and 60% for patients with invasion [5]. Previously, lesions in the gallbladder, if smaller in size with no alarming characteristics, were considered as benign polypoidal lesions, or if forming mass-like lesions with aggressive clinico-pathological features, were considered as gallbladder carcinoma and were treated aggressively. This in-between spectrum lesion, ICPN, is a relatively new entity with no defined line of management due to the scarcity of cases in the literature [6].

Koike et al. reported a case where they followed up an asymptomatic patient with a 3 mm gallbladder polyp signifying the natural history of ICPN [7]. After two years of ultrasonographic follow-up, the polyp progressed to a markedly enlarged polypoidal lesion with a postoperative histopathological diagnosis of ICPN with high-grade intraepithelial neoplasm of gastric type. This emphasizes the importance of regular follow-up of suspected lesions in the gallbladder.

Recently, many case reports have been published regarding preoperative suspicion of ICPN with mass-like lesions in the gallbladder [8,9]. Authors in these reports focused on preoperative imaging characteristics of lesions on CT scans and magnetic resonance imaging (MRI) scans. Treatment offered was a laparoscopic cholecystectomy, and postoperative histopathology revealed ICPN.

Though ICPN is a premalignant lesion, it is distinct from gallbladder carcinomas. Akita et al. performed a clinic-pathological and exome sequencing study comparing these entities [6]. Gross mucin hypersecretion was detected in 43% of ICPN patients as compared to 2-4% in gallbladder carcinomas. All patients with ICPN lacked lymphovascular invasion and nodal metastasis, while these features were occasionally present in gallbladder cancers. ICPNs were less advanced and were recurrence-free as compared to gallbladder neoplasms. Serine/threonine kinase 11 (STK11), cadherin-associated protein (CTNNB1), and adenomatous polyposis coli (APC) genes were being identified as major driver genes for ICPN.

On the other end of the spectrum, Suda et al. published a case report of an 83-year-old patient presenting with multiple liver metastases with raised CEA and CA 19-9 with no known primary tumor [3]. The patient succumbed to illness, but the autopsy revealed a 1 cm fragile papillary tumor at the fundus of the gallbladder. A pathological diagnosis of ICPN with an associated invasive carcinoma was established.

It is apparent from the above-mentioned case reports that ICPN as a disease entity is less aggressive than carcinoma of the gallbladder, but occasionally, the presence of invasive carcinoma in postoperative histopathology specimens raises concerns [4]. As in most surgeries, a complete hepatoduodenal lymphadenectomy has not been performed, and follow-up of these patients is for limited time intervals only; occult metastasis in lymph nodes could not be ruled out.

Nakayama et al. emphasized the pathological evaluation of lymph node metastasis in ICPN patients for determining prognosis [10]. In our patient, all lymph nodes were negative for malignancy in histopathology. In our opinion, if any aggressive features are seen preoperatively, such as a symptomatic patient (pain, jaundice, weight loss, anorexia), raised tumor markers (CEA, CA 19-9), suspicious lymph nodes in imaging, or a large lesion size, radical cholecystectomy with standard lymphadenectomy could be a more appropriate treatment option as compared to simple cholecystectomy, especially in countries where stringent follow-up of patients could not be done due to limited resources. Invasive carcinoma of the gallbladder most often provides a single surgical chance for a cure; if the opportunity is missed, the outcome would not be good for patients.

## Conclusions

ICPN, being a preinvasive gallbladder neoplasm, is less invasive and has a better prognosis than gallbladder carcinoma. The presence of an invasive component at the time of preoperative detection cannot be determined. Invasive carcinomas may be associated with lymph nodal metastasis. Standardized guidelines are required for preoperative evaluation, investigations, and surgical planning in patients diagnosed with ICPN. 
